# {6,6′-Dimeth­oxy-2,2′-[(cyclo­hexane-1,2-di­yl)bis­(nitriliomethyl­idyne)]diphenolato}trinitratolanthanum(III) methanol monosolvate

**DOI:** 10.1107/S1600536810033453

**Published:** 2010-08-28

**Authors:** Peng Chen, Yan Bao, Peng-Fei Yan, Guang-Ming Li

**Affiliations:** a School of Chemistry and Materials Science, Heilongjiang University, Harbin 150080, People’s Republic of China

## Abstract

In the title mononuclear complex, [La(NO_3_)_3_(C_22_H_26_N_2_O_4_)]·CH_3_OH, the La^III^ ion is coordinated by three bidentate nitrate counter-ions and one zwitterionic 6,6′-dimeth­oxy-2,2′-[(cyclo­hexane-1,2- di­yl)bis­(nitriliomethyl­idyne)]diphenolate ligand through two phenolate and two meth­oxy O atoms, while the protonated N atoms remain uncoordinated. H atoms located on the two N atoms are involved in intra­molecular hydrogen bonds with the deprotonated phenol O atoms, indicating that proton migration occurs during the lanthanum complexation.

## Related literature

For the preparation of the ligand, see: Koner *et al.* (2005 ▶). For a related structure, see: Yan *et al.* (2009 ▶).
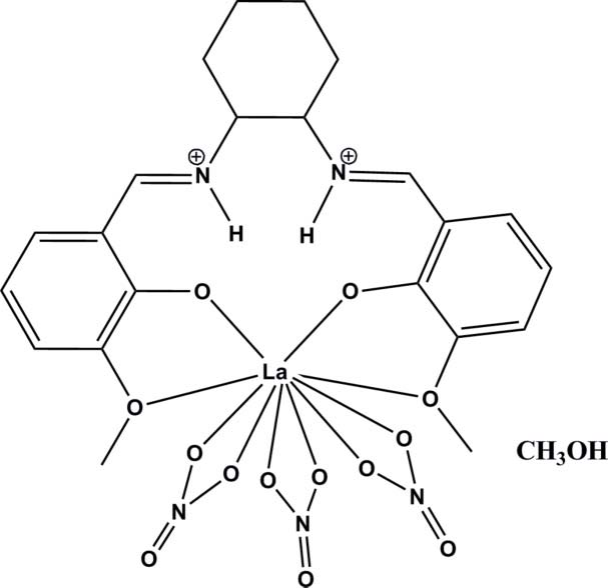


## Experimental

### 

#### Crystal data

[La(NO_3_)_3_(C_22_H_26_N_2_O_4_)]·CH_4_O
*M* *_r_* = 739.43
Triclinic, 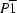

*a* = 9.7809 (4) Å
*b* = 12.8783 (5) Å
*c* = 13.0904 (5) Å
α = 79.374 (1)°
β = 68.743 (1)°
γ = 82.270 (1)°
*V* = 1506.22 (10) Å^3^
*Z* = 2
Mo *K*α radiation
μ = 1.49 mm^−1^
*T* = 293 K
0.23 × 0.20 × 0.16 mm

#### Data collection

Rigaku R-AXIS RAPID diffractometer
Absorption correction: multi-scan (*ABSCOR*; Higashi, 1995 ▶) *T*_min_ = 0.725, *T*_max_ = 0.796
10874 measured reflections
7145 independent reflections
6526 reflections with *I* > 2σ(*I*)
*R*_int_ = 0.010

#### Refinement

*R*[*F* ^2^ > 2σ(*F* ^2^)] = 0.033
*wR*(*F* ^2^) = 0.092
*S* = 1.01
7145 reflections
389 parameters
38 restraints
H-atom parameters constrained
Δρ_max_ = 0.97 e Å^−3^
Δρ_min_ = −0.77 e Å^−3^

### 

Data collection: *RAPID-AUTO* (Rigaku, 1998 ▶); cell refinement: *RAPID-AUTO*; data reduction: *CrystalStructure* (Rigaku/MSC, 2002 ▶); program(s) used to solve structure: *SHELXS97* (Sheldrick, 2008 ▶); program(s) used to refine structure: *SHELXL97* (Sheldrick, 2008 ▶); molecular graphics: *SHELXTL* (Sheldrick, 2008 ▶); software used to prepare material for publication: *SHELXL97*.

## Supplementary Material

Crystal structure: contains datablocks global, I. DOI: 10.1107/S1600536810033453/vm2037sup1.cif

Structure factors: contains datablocks I. DOI: 10.1107/S1600536810033453/vm2037Isup2.hkl

Additional supplementary materials:  crystallographic information; 3D view; checkCIF report

## Figures and Tables

**Table 1:** Hydrogen-bond geometry (Å, °)

*D*—H⋯*A*	*D*—H	H⋯*A*	*D*⋯*A*	*D*—H⋯*A*
N1—H1*A*⋯O1	0.86	1.86	2.562 (3)	137
N2—H2*A*⋯O3	0.86	1.89	2.592 (3)	138
O14—H14⋯O5^i^	0.84	2.40	2.981 (7)	127

Symmetry code: (i) 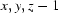.

**Fig. 1.**
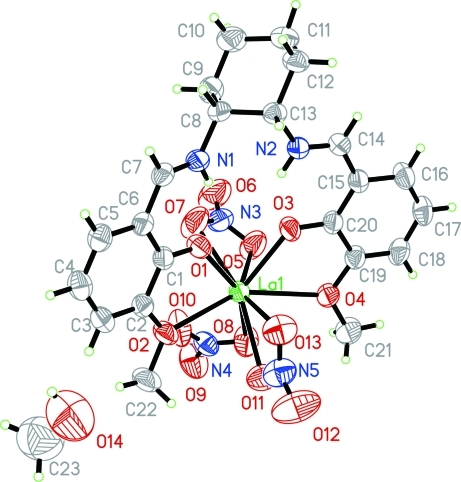
The molecular structure of the title compound, showing 50% probability displacement ellipsoids.

## supplementary crystallographic information

### Comment

Salen-type ligands are capable to incorporate lanthanide ions and to form complexes in the outer coordination site. Such complexes are potentially used in magnets and optics. In continuation of our studies of salen-type lanthanide complexes (Yan *et al.*, 2009), we present here the crystal structure of the title compound. As shown in Fig. 1, the La^III^ ion is coordinated to three bidentate nitrate counterions and one ligand that utilizes two hydroxyl oxygen atoms and two methoxyl oxygen atoms, while the nitrogen atoms remain uncoordinated (Koner *et al.*, 2005). The La^III^ ion presents a narrow spread in La—O bond distances [2.406 (2)–2.787 (2) Å], and the La—N bond distances are relatively longer [3.034 (4) and 3.048 (3) Å]. Hydrogen atoms located on the two nitrogen atoms are involved in intramolecular hydrogen bonds with the deprotonated phenol oxygen atoms (Table 1), which might contribute to the stability of the whole structure.

### Experimental

To a CH_2_Cl_2_ solution (5 ml) of H_2_L (0.0382 g, 0.1 mmol) under stirring was slowly added a MeCN solution (5 ml) of La(NO_3_)_3_.6H_2_O (0.0433 g, 0.1 mmol) at room temperature. The diethylether was allowed to diffuse slowly into the filtrate at room temperature. The light yellow crystals were obtained in one week. (H_2_L)La(NO_3_)_3_.CH_3_OH. Yield: 0.0426 g (57.4 wt%). Elemental Anal. Calc. for C_23_H_30_N_5_O_14_La: C, 37.36; H, 4.09; N, 9.47 wt%, Found: C, 37.21; H, 4.15; N, 9.44 wt%.

### Refinement

H atoms bound to C atoms were placed in calculated positions and treated as riding on their parent atoms, with C—H = 0.93 Å (aromatic C), C—H = 0.97Å (methylene C), and with *U*_iso_(H) = 1.2Ueq(C) or C—H = 0.96 Å (methly C) and with *U*_iso_(H) = 1.5Ueq(C). The N-bound H atoms were initially located in a difference Fourier map and they were refined with N—H=0.85 Å. H atoms bound to O atoms were found from the Fourier difference map, and the distance is refined in the normal range with *U*_iso_(H) = 1.5 *U*_eq_(O).

### Crystal data

**Table:**

[La(NO_3_)_3_(C_22_H_26_N_2_O_4_)]·CH_4_O	*Z* = 2
*M**_r_* = 739.43	*F*(000) = 744
Triclinic, *P*1	*D*_x_ = 1.630 Mg m^−^^3^
Hall symbol: -P 1	Mo *K*α radiation, λ = 0.71073 Å
*a* = 9.7809 (4) Å	Cell parameters from 6687 reflections
*b* = 12.8783 (5) Å	θ = 2.7–28.3°
*c* = 13.0904 (5) Å	µ = 1.49 mm^−^^1^
α = 79.374 (1)°	*T* = 293 K
β = 68.743 (1)°	Block, yellow
γ = 82.270 (1)°	0.23 × 0.20 × 0.16 mm
*V* = 1506.22 (10) Å^3^	

### Data collection

**Table:**

Rigaku R-AXIS RAPID diffractometer	7145 independent reflections
Radiation source: fine-focus sealed tube	6526 reflections with *I* > 2σ(*I*)
graphite	*R*_int_ = 0.010
Detector resolution: 10.000 pixels mm^-1^	θ_max_ = 28.3°, θ_min_ = 2.7°
ω scan	*h* = −12→12
Absorption correction: multi-scan (*ABSCOR*; Higashi, 1995)	*k* = −17→10
*T*_min_ = 0.725, *T*_max_ = 0.796	*l* = −17→17
10874 measured reflections	

### Refinement

**Table:**

Refinement on *F*^2^	Primary atom site location: structure-invariant direct methods
Least-squares matrix: full	Secondary atom site location: difference Fourier map
*R*[*F*^2^ > 2σ(*F*^2^)] = 0.033	Hydrogen site location: inferred from neighbouring sites
*wR*(*F*^2^) = 0.092	H-atom parameters constrained
*S* = 1.01	*w* = 1/[σ^2^(*F*_o_^2^) + (0.052*P*)^2^ + 1.3148*P*] where *P* = (*F*_o_^2^ + 2*F*_c_^2^)/3
7145 reflections	(Δ/σ)_max_ = 0.011
389 parameters	Δρ_max_ = 0.97 e Å^−^^3^
38 restraints	Δρ_min_ = −0.77 e Å^−^^3^

### Special details

**Table:**

Geometry. All e.s.d.'s (except the e.s.d. in the dihedral angle between two l.s. planes) are estimated using the full covariance matrix. The cell e.s.d.'s are taken into account individually in the estimation of e.s.d.'s in distances, angles and torsion angles; correlations between e.s.d.'s in cell parameters are only used when they are defined by crystal symmetry. An approximate (isotropic) treatment of cell e.s.d.'s is used for estimating e.s.d.'s involving l.s. planes.
Refinement. Refinement of *F*^2^ against ALL reflections. The weighted *R*-factor *wR* and goodness of fit *S* are based on *F*^2^, conventional *R*-factors *R* are based on *F*, with *F* set to zero for negative *F*^2^. The threshold expression of *F*^2^ > σ(*F*^2^) is used only for calculating *R*-factors(gt) *etc*. and is not relevant to the choice of reflections for refinement. *R*-factors based on *F*^2^ are statistically about twice as large as those based on *F*, and *R*- factors based on ALL data will be even larger.

### Fractional atomic coordinates and isotropic or equivalent isotropic displacement parameters (Å^2^)

**Table:**

	*x*	*y*	*z*	*U*_iso_*/*U*_eq_	
La1	0.172411 (19)	0.227443 (12)	0.702241 (13)	0.04350 (7)	
C1	0.2772 (3)	0.3560 (2)	0.4366 (2)	0.0404 (6)	
C2	0.2940 (3)	0.2558 (2)	0.4018 (3)	0.0419 (6)	
C3	0.3415 (3)	0.2462 (3)	0.2918 (3)	0.0482 (7)	
H3A	0.3528	0.1797	0.2703	0.058*	
C4	0.3735 (4)	0.3370 (3)	0.2111 (3)	0.0533 (8)	
H4A	0.4072	0.3299	0.1364	0.064*	
C5	0.3555 (4)	0.4351 (3)	0.2413 (3)	0.0483 (7)	
H5A	0.3755	0.4946	0.1873	0.058*	
C6	0.3065 (3)	0.4467 (2)	0.3548 (2)	0.0392 (6)	
C7	0.2909 (3)	0.5487 (2)	0.3870 (2)	0.0414 (6)	
H7A	0.3062	0.6077	0.3324	0.050*	
C8	0.2394 (3)	0.6651 (2)	0.5270 (2)	0.0394 (6)	
H8A	0.2616	0.7200	0.4618	0.047*	
C9	0.3472 (4)	0.6692 (3)	0.5844 (3)	0.0532 (8)	
H9A	0.4468	0.6553	0.5346	0.064*	
H9B	0.3286	0.6153	0.6492	0.064*	
C10	0.3307 (4)	0.7787 (3)	0.6190 (4)	0.0651 (10)	
H10A	0.3964	0.7798	0.6593	0.078*	
H10B	0.3593	0.8314	0.5532	0.078*	
C11	0.1746 (4)	0.8071 (3)	0.6911 (3)	0.0562 (8)	
H11A	0.1666	0.8794	0.7052	0.067*	
H11B	0.1512	0.7610	0.7617	0.067*	
C12	0.0645 (4)	0.7966 (2)	0.6370 (3)	0.0497 (7)	
H12A	0.0780	0.8508	0.5727	0.060*	
H12B	−0.0343	0.8086	0.6888	0.060*	
C13	0.0799 (3)	0.6883 (2)	0.6012 (3)	0.0408 (6)	
H13A	0.0162	0.6905	0.5578	0.049*	
C14	−0.0445 (3)	0.6076 (2)	0.7970 (3)	0.0451 (6)	
H14A	−0.0765	0.6743	0.8180	0.054*	
C15	−0.0888 (3)	0.5169 (3)	0.8781 (3)	0.0450 (6)	
C16	−0.1899 (4)	0.5304 (3)	0.9847 (3)	0.0554 (8)	
H16A	−0.2188	0.5981	1.0038	0.066*	
C17	−0.2449 (4)	0.4451 (3)	1.0591 (3)	0.0600 (9)	
H17A	−0.3119	0.4548	1.1289	0.072*	
C18	−0.2024 (4)	0.3425 (3)	1.0326 (3)	0.0544 (8)	
H18A	−0.2434	0.2848	1.0838	0.065*	
C19	−0.1002 (3)	0.3270 (3)	0.9312 (3)	0.0453 (6)	
C20	−0.0405 (3)	0.4140 (2)	0.8507 (3)	0.0446 (6)	
C21	−0.1055 (5)	0.1379 (3)	0.9726 (4)	0.0780 (13)	
H21A	−0.1838	0.1597	1.0361	0.117*	
H21B	−0.0289	0.0979	0.9957	0.117*	
H21C	−0.1425	0.0948	0.9374	0.117*	
C22	0.2813 (5)	0.0690 (3)	0.4624 (4)	0.0691 (11)	
H22A	0.3126	0.0721	0.3835	0.104*	
H22B	0.1898	0.0361	0.4966	0.104*	
H22C	0.3544	0.0283	0.4891	0.104*	
C23	0.2873 (10)	0.0899 (7)	0.0851 (7)	0.144 (3)	
H29	0.3782	0.1074	0.0269	0.216*	
H30	0.2505	0.0311	0.0699	0.216*	
H31	0.3039	0.0710	0.1544	0.216*	
N1	0.2565 (3)	0.56252 (19)	0.4883 (2)	0.0435 (5)	
H1A	0.2423	0.5070	0.5376	0.052*	
N2	0.0379 (3)	0.6013 (2)	0.6959 (2)	0.0444 (5)	
H2A	0.0732	0.5387	0.6813	0.053*	
N3	0.3925 (4)	0.2989 (3)	0.7882 (3)	0.0685 (9)	
N4	0.3331 (4)	0.0205 (2)	0.7668 (3)	0.0568 (7)	
N5	−0.0827 (4)	0.1433 (3)	0.6708 (3)	0.0700 (9)	
O1	0.2366 (3)	0.36209 (17)	0.54227 (18)	0.0551 (6)	
O2	0.2617 (3)	0.17450 (17)	0.4894 (2)	0.0548 (6)	
O3	0.0536 (3)	0.39767 (17)	0.75338 (19)	0.0569 (6)	
O4	−0.0473 (3)	0.23020 (17)	0.89550 (19)	0.0536 (6)	
O5	0.2776 (4)	0.2560 (3)	0.8491 (2)	0.0817 (9)	
O6	0.4763 (5)	0.3239 (4)	0.8270 (3)	0.1080 (14)	
O7	0.4123 (4)	0.3163 (3)	0.6873 (3)	0.0898 (10)	
O8	0.2108 (3)	0.0497 (2)	0.8293 (3)	0.0755 (8)	
O9	0.4015 (4)	−0.0591 (3)	0.7944 (3)	0.0891 (10)	
O10	0.3813 (4)	0.0756 (3)	0.6739 (3)	0.0857 (10)	
O11	0.0096 (3)	0.0836 (2)	0.7053 (3)	0.0698 (7)	
O12	−0.1833 (5)	0.1093 (4)	0.6569 (4)	0.1176 (16)	
O13	−0.0650 (3)	0.2414 (2)	0.6515 (3)	0.0732 (8)	
O14	0.1852 (7)	0.1766 (5)	0.0913 (5)	0.164 (2)	
H14	0.2229	0.2291	0.0457	0.246*	

### Atomic displacement parameters (Å^2^)

**Table:**

	*U*^11^	*U*^22^	*U*^33^	*U*^12^	*U*^13^	*U*^23^
La1	0.05179 (11)	0.03034 (9)	0.03740 (10)	−0.00171 (7)	−0.00476 (7)	−0.00129 (6)
C1	0.0391 (13)	0.0398 (14)	0.0375 (14)	−0.0013 (11)	−0.0082 (11)	−0.0056 (11)
C2	0.0384 (13)	0.0390 (14)	0.0446 (15)	0.0008 (11)	−0.0106 (11)	−0.0073 (12)
C3	0.0464 (15)	0.0504 (16)	0.0498 (17)	0.0057 (13)	−0.0171 (13)	−0.0183 (14)
C4	0.0576 (18)	0.065 (2)	0.0374 (15)	−0.0006 (16)	−0.0155 (14)	−0.0125 (15)
C5	0.0493 (16)	0.0564 (18)	0.0371 (14)	−0.0064 (14)	−0.0143 (12)	−0.0012 (13)
C6	0.0370 (13)	0.0406 (14)	0.0367 (13)	−0.0040 (11)	−0.0099 (11)	−0.0028 (11)
C7	0.0413 (13)	0.0408 (15)	0.0377 (14)	−0.0076 (11)	−0.0101 (11)	0.0005 (11)
C8	0.0426 (14)	0.0314 (12)	0.0422 (14)	−0.0062 (11)	−0.0130 (11)	−0.0013 (11)
C9	0.0431 (15)	0.059 (2)	0.059 (2)	−0.0016 (14)	−0.0205 (14)	−0.0083 (16)
C10	0.059 (2)	0.064 (2)	0.085 (3)	−0.0135 (18)	−0.0314 (19)	−0.020 (2)
C11	0.068 (2)	0.0455 (17)	0.062 (2)	−0.0092 (15)	−0.0260 (17)	−0.0141 (15)
C12	0.0541 (17)	0.0343 (14)	0.0599 (19)	0.0000 (13)	−0.0207 (15)	−0.0055 (13)
C13	0.0413 (13)	0.0342 (13)	0.0475 (15)	−0.0023 (11)	−0.0177 (12)	−0.0033 (11)
C14	0.0431 (14)	0.0388 (14)	0.0508 (17)	0.0008 (12)	−0.0128 (13)	−0.0105 (13)
C15	0.0427 (14)	0.0448 (16)	0.0417 (15)	−0.0012 (12)	−0.0074 (12)	−0.0082 (12)
C16	0.0562 (18)	0.058 (2)	0.0438 (17)	0.0057 (15)	−0.0068 (14)	−0.0160 (15)
C17	0.0538 (18)	0.074 (2)	0.0383 (16)	0.0018 (17)	−0.0002 (14)	−0.0112 (16)
C18	0.0495 (16)	0.061 (2)	0.0397 (16)	−0.0070 (15)	−0.0043 (13)	0.0031 (14)
C19	0.0432 (14)	0.0440 (15)	0.0403 (15)	−0.0018 (12)	−0.0074 (12)	−0.0011 (12)
C20	0.0411 (14)	0.0410 (15)	0.0416 (15)	−0.0011 (12)	−0.0045 (12)	−0.0034 (12)
C21	0.089 (3)	0.0441 (19)	0.069 (3)	−0.0140 (19)	0.004 (2)	0.0125 (18)
C22	0.095 (3)	0.0348 (16)	0.068 (2)	−0.0024 (17)	−0.015 (2)	−0.0143 (16)
C23	0.162 (4)	0.146 (4)	0.137 (4)	−0.019 (3)	−0.061 (3)	−0.028 (3)
N1	0.0522 (13)	0.0331 (11)	0.0386 (12)	−0.0058 (10)	−0.0098 (10)	0.0001 (9)
N2	0.0450 (12)	0.0350 (12)	0.0454 (13)	−0.0035 (10)	−0.0069 (10)	−0.0048 (10)
N3	0.076 (2)	0.065 (2)	0.060 (2)	−0.0281 (17)	−0.0106 (16)	−0.0104 (16)
N4	0.0677 (18)	0.0447 (15)	0.0576 (17)	0.0093 (13)	−0.0234 (15)	−0.0126 (13)
N5	0.086 (2)	0.069 (2)	0.0593 (19)	−0.0215 (19)	−0.0333 (18)	0.0070 (16)
O1	0.0816 (16)	0.0351 (10)	0.0339 (10)	−0.0054 (10)	−0.0034 (10)	−0.0029 (8)
O2	0.0737 (15)	0.0334 (10)	0.0478 (12)	0.0012 (10)	−0.0102 (11)	−0.0093 (9)
O3	0.0665 (14)	0.0347 (11)	0.0437 (12)	−0.0009 (10)	0.0087 (10)	−0.0023 (9)
O4	0.0587 (13)	0.0373 (11)	0.0471 (12)	−0.0066 (10)	−0.0015 (10)	0.0048 (9)
O5	0.0813 (19)	0.109 (3)	0.0504 (15)	−0.0419 (19)	−0.0067 (14)	−0.0084 (16)
O6	0.104 (3)	0.144 (4)	0.089 (2)	−0.064 (3)	−0.028 (2)	−0.020 (2)
O7	0.087 (2)	0.117 (3)	0.0558 (17)	−0.048 (2)	−0.0052 (15)	0.0010 (17)
O8	0.0697 (17)	0.0567 (15)	0.0721 (18)	0.0137 (13)	−0.0071 (14)	0.0099 (13)
O9	0.094 (2)	0.073 (2)	0.085 (2)	0.0350 (17)	−0.0314 (18)	−0.0055 (17)
O10	0.084 (2)	0.082 (2)	0.0566 (16)	0.0293 (16)	−0.0019 (14)	−0.0002 (15)
O11	0.0806 (18)	0.0499 (14)	0.0781 (19)	−0.0116 (13)	−0.0315 (15)	0.0058 (13)
O12	0.138 (4)	0.110 (3)	0.139 (4)	−0.052 (3)	−0.093 (3)	0.021 (3)
O13	0.0836 (19)	0.0607 (17)	0.0798 (19)	−0.0035 (14)	−0.0426 (16)	0.0065 (14)
O14	0.181 (3)	0.165 (3)	0.146 (3)	0.001 (3)	−0.052 (2)	−0.044 (3)

### Geometric parameters (Å, °)

**Table:**

La1—O1	2.406 (2)	C12—H12B	0.9700
La1—O3	2.428 (2)	C13—N2	1.484 (4)
La1—O5	2.586 (3)	C13—H13A	0.9800
La1—O11	2.587 (3)	C14—N2	1.287 (4)
La1—O10	2.604 (3)	C14—C15	1.423 (4)
La1—O13	2.613 (3)	C14—H14A	0.9300
La1—O8	2.639 (3)	C15—C20	1.411 (4)
La1—O4	2.663 (2)	C15—C16	1.414 (4)
La1—O7	2.676 (3)	C16—C17	1.352 (5)
La1—O2	2.787 (2)	C16—H16A	0.9300
La1—N5	3.034 (4)	C17—C18	1.398 (5)
La1—N4	3.048 (3)	C17—H17A	0.9300
C1—O1	1.308 (4)	C18—C19	1.372 (4)
C1—C6	1.415 (4)	C18—H18A	0.9300
C1—C2	1.416 (4)	C19—O4	1.381 (4)
C2—C3	1.367 (4)	C19—C20	1.415 (4)
C2—O2	1.376 (4)	C20—O3	1.308 (4)
C3—C4	1.409 (5)	C21—O4	1.441 (4)
C3—H3A	0.9300	C21—H21A	0.9600
C4—C5	1.364 (5)	C21—H21B	0.9600
C4—H4A	0.9300	C21—H21C	0.9600
C5—C6	1.415 (4)	C22—O2	1.437 (4)
C5—H5A	0.9300	C22—H22A	0.9600
C6—C7	1.425 (4)	C22—H22B	0.9600
C7—N1	1.285 (4)	C22—H22C	0.9600
C7—H7A	0.9300	C23—O14	1.388 (7)
C8—N1	1.468 (4)	C23—H29	0.9600
C8—C9	1.510 (4)	C23—H30	0.9600
C8—C13	1.533 (4)	C23—H31	0.9600
C8—H8A	0.9800	N1—H1A	0.8600
C9—C10	1.530 (5)	N2—H2A	0.8600
C9—H9A	0.9700	N3—O6	1.211 (5)
C9—H9B	0.9700	N3—O7	1.244 (5)
C10—C11	1.513 (5)	N3—O5	1.249 (4)
C10—H10A	0.9700	N4—O9	1.218 (4)
C10—H10B	0.9700	N4—O8	1.235 (4)
C11—C12	1.518 (5)	N4—O10	1.249 (4)
C11—H11A	0.9700	N5—O12	1.213 (5)
C11—H11B	0.9700	N5—O11	1.261 (5)
C12—C13	1.523 (4)	N5—O13	1.264 (5)
C12—H12A	0.9700	O14—H14	0.8416
			
O1—La1—O3	70.12 (7)	C11—C10—C9	111.9 (3)
O1—La1—O5	112.10 (10)	C11—C10—H10A	109.2
O3—La1—O5	77.05 (11)	C9—C10—H10A	109.2
O1—La1—O11	117.56 (9)	C11—C10—H10B	109.2
O3—La1—O11	118.33 (9)	C9—C10—H10B	109.2
O5—La1—O11	130.32 (10)	H10A—C10—H10B	107.9
O1—La1—O10	108.80 (9)	C10—C11—C12	111.8 (3)
O3—La1—O10	155.24 (12)	C10—C11—H11A	109.3
O5—La1—O10	80.89 (12)	C12—C11—H11A	109.3
O11—La1—O10	84.70 (11)	C10—C11—H11B	109.3
O1—La1—O13	80.42 (9)	C12—C11—H11B	109.3
O3—La1—O13	77.22 (10)	H11A—C11—H11B	107.9
O5—La1—O13	145.03 (11)	C11—C12—C13	112.8 (3)
O11—La1—O13	48.74 (9)	C11—C12—H12A	109.0
O10—La1—O13	127.48 (12)	C13—C12—H12A	109.0
O1—La1—O8	156.29 (9)	C11—C12—H12B	109.0
O3—La1—O8	129.69 (9)	C13—C12—H12B	109.0
O5—La1—O8	67.36 (12)	H12A—C12—H12B	107.8
O11—La1—O8	67.92 (10)	N2—C13—C12	113.2 (3)
O10—La1—O8	47.55 (9)	N2—C13—C8	109.2 (2)
O13—La1—O8	114.12 (10)	C12—C13—C8	109.2 (2)
O1—La1—O4	129.58 (7)	N2—C13—H13A	108.4
O3—La1—O4	61.66 (7)	C12—C13—H13A	108.4
O5—La1—O4	71.42 (9)	C8—C13—H13A	108.4
O11—La1—O4	76.31 (9)	N2—C14—C15	122.9 (3)
O10—La1—O4	121.04 (8)	N2—C14—H14A	118.5
O13—La1—O4	75.68 (9)	C15—C14—H14A	118.5
O8—La1—O4	73.68 (8)	C20—C15—C16	120.0 (3)
O1—La1—O7	70.19 (10)	C20—C15—C14	120.5 (3)
O3—La1—O7	82.23 (11)	C16—C15—C14	119.4 (3)
O5—La1—O7	47.34 (9)	C17—C16—C15	120.2 (3)
O11—La1—O7	159.25 (12)	C17—C16—H16A	119.9
O10—La1—O7	74.55 (13)	C15—C16—H16A	119.9
O13—La1—O7	148.50 (11)	C16—C17—C18	120.9 (3)
O8—La1—O7	97.37 (11)	C16—C17—H17A	119.5
O4—La1—O7	114.71 (9)	C18—C17—H17A	119.5
O1—La1—O2	59.31 (7)	C19—C18—C17	120.1 (3)
O3—La1—O2	124.53 (8)	C19—C18—H18A	120.0
O5—La1—O2	141.26 (9)	C17—C18—H18A	120.0
O11—La1—O2	71.75 (9)	C18—C19—O4	125.9 (3)
O10—La1—O2	68.96 (9)	C18—C19—C20	120.8 (3)
O13—La1—O2	73.57 (9)	O4—C19—C20	113.3 (3)
O8—La1—O2	105.25 (9)	O3—C20—C15	122.1 (3)
O4—La1—O2	145.47 (8)	O3—C20—C19	119.9 (3)
O7—La1—O2	99.75 (9)	C15—C20—C19	117.9 (3)
O1—La1—N5	99.24 (9)	O4—C21—H21A	109.5
O3—La1—N5	98.20 (10)	O4—C21—H21B	109.5
O5—La1—N5	143.84 (9)	H21A—C21—H21B	109.5
O11—La1—N5	24.29 (9)	O4—C21—H21C	109.5
O10—La1—N5	106.26 (12)	H21A—C21—H21C	109.5
O13—La1—N5	24.45 (9)	H21B—C21—H21C	109.5
O8—La1—N5	91.04 (10)	O2—C22—H22A	109.5
O4—La1—N5	74.87 (9)	O2—C22—H22B	109.5
O7—La1—N5	168.70 (10)	H22A—C22—H22B	109.5
O2—La1—N5	70.62 (9)	O2—C22—H22C	109.5
O1—La1—N4	132.60 (9)	H22A—C22—H22C	109.5
O3—La1—N4	146.68 (9)	H22B—C22—H22C	109.5
O5—La1—N4	71.57 (11)	O14—C23—H29	109.5
O11—La1—N4	76.25 (9)	O14—C23—H30	109.5
O10—La1—N4	23.91 (9)	H29—C23—H30	109.5
O13—La1—N4	124.87 (9)	O14—C23—H31	109.5
O8—La1—N4	23.70 (8)	H29—C23—H31	109.5
O4—La1—N4	97.16 (8)	H30—C23—H31	109.5
O7—La1—N4	84.69 (11)	C7—N1—C8	125.6 (2)
O2—La1—N4	87.83 (7)	C7—N1—H1A	117.2
N5—La1—N4	100.47 (10)	C8—N1—H1A	117.2
O1—C1—C6	122.1 (3)	C14—N2—C13	128.3 (3)
O1—C1—C2	119.6 (3)	C14—N2—H2A	115.8
C6—C1—C2	118.3 (3)	C13—N2—H2A	115.8
C3—C2—O2	126.5 (3)	O6—N3—O7	123.0 (4)
C3—C2—C1	121.1 (3)	O6—N3—O5	120.9 (4)
O2—C2—C1	112.4 (3)	O7—N3—O5	116.0 (3)
C2—C3—C4	120.0 (3)	O9—N4—O8	121.2 (3)
C2—C3—H3A	120.0	O9—N4—O10	122.1 (3)
C4—C3—H3A	120.0	O8—N4—O10	116.7 (3)
C5—C4—C3	120.7 (3)	O9—N4—La1	176.5 (3)
C5—C4—H4A	119.6	O8—N4—La1	59.21 (17)
C3—C4—H4A	119.6	O10—N4—La1	57.69 (17)
C4—C5—C6	120.1 (3)	O12—N5—O11	122.3 (4)
C4—C5—H5A	119.9	O12—N5—O13	121.3 (4)
C6—C5—H5A	119.9	O11—N5—O13	116.3 (3)
C1—C6—C5	119.8 (3)	O12—N5—La1	179.1 (4)
C1—C6—C7	119.7 (3)	O11—N5—La1	57.56 (19)
C5—C6—C7	120.5 (3)	O13—N5—La1	58.80 (19)
N1—C7—C6	122.8 (3)	C1—O1—La1	131.06 (19)
N1—C7—H7A	118.6	C2—O2—C22	116.7 (3)
C6—C7—H7A	118.6	C2—O2—La1	117.55 (17)
N1—C8—C9	111.1 (2)	C22—O2—La1	125.7 (2)
N1—C8—C13	110.4 (2)	C20—O3—La1	125.86 (19)
C9—C8—C13	112.1 (3)	C19—O4—C21	116.4 (3)
N1—C8—H8A	107.7	C19—O4—La1	117.93 (17)
C9—C8—H8A	107.7	C21—O4—La1	125.3 (2)
C13—C8—H8A	107.7	N3—O5—La1	100.4 (2)
C8—C9—C10	109.5 (3)	N3—O7—La1	96.1 (2)
C8—C9—H9A	109.8	N4—O8—La1	97.1 (2)
C10—C9—H9A	109.8	N4—O10—La1	98.4 (2)
C8—C9—H9B	109.8	N5—O11—La1	98.1 (2)
C10—C9—H9B	109.8	N5—O13—La1	96.8 (2)
H9A—C9—H9B	108.2	C23—O14—H14	111.0

### Hydrogen-bond geometry (Å, °)

**Table:**

*D*—H···*A*	*D*—H	H···*A*	*D*···*A*	*D*—H···*A*
N1—H1A···O1	0.86	1.86	2.562 (3)	137.
N2—H2A···O3	0.86	1.89	2.592 (3)	138.
O14—H14···O5^i^	0.84	2.40	2.981 (7)	127.

Symmetry codes: (i) *x*, *y*, *z*−1.
